# Creating an Intercultural User-Centric Design for a Digital Sexual Health Education App for Young Women in Resource-Poor Regions of Kenya: Qualitative Self-Extended Double Diamond Model for Requirements Engineering Analysis

**DOI:** 10.2196/50304

**Published:** 2023-11-03

**Authors:** Clarissa Soehnchen, Annika Rietz, Vera Weirauch, Sven Meister, Maike Henningsen

**Affiliations:** 1 HealthCare Department Fraunhofer Institute for Software and System Engineering Dortmund Germany; 2 Health Care Informatics, Faculty of Health/School of Medicine Witten/Herdecke University Witten Germany

**Keywords:** sexual health information, sexual health education, sub-Saharan Africa, women, semistructured interviews, requirements engineering analysis, user-centered design, youth, slum, health education, sexual health, digital health, stigmatization, reproductive health services

## Abstract

**Background:**

The stigmatization around sexual health due to culture, religious traditions, and norms leads to myths and a lack of available information and resources related to universal access to sexual and reproductive health services. Additional sexual health education not being part of the Kenya school curriculum leads to insufficient access to knowledge about safe contraception, menstruation, and female genital mutilation. A digital app could support and provide education and information for universal equal access, addressing United Nations Sustainable Development Goals 3, 4, and 5.

**Objective:**

The study targeted the requirements for establishing a reusable framework to develop a successful accessible web-based sexual health education app and the behavioral intention to use it to obtain sexual health information by mainly young women in Kenya.

**Methods:**

The double diamond model, with a problem room including the discover and define phases, enriched with cultural aspects and modeled to a self-expanded intercultural research model was used in a user-centered design thinking approach to develop a framework for requirements engineering analysis. For problem identification, semistructured pilot phase interviews based on Consolidated Criteria for Reporting Qualitative Research guidelines were conducted, followed by expert interviews for qualitative content analysis. A sample size of 12 pilot phase interviews and 5 expert interviews was determined using data saturation. The responses were coded and analyzed according to the affinity mapping method.

**Results:**

The requirements engineering analysis showed potential enablers of and barriers to the use of a digital sexual health education app. Through this qualitative study, a conservative cultural background, classic text communication, and the influence of social affiliation within society were identified as barriers, which should be enhanced through visual and auditory channels as well as a fictional character in the app.

**Conclusions:**

The developed intercultural research model provides an impetus to providing digital sexual health education, integrating culture-specific aspects in the design process, while focusing on cultural and religious stigmata. The reusable framework enables identifying and overcoming hurdles in providing information about taboo and intimate topics. The overall use of online education tools focusing on intimate topics is correlated with accessibility and understanding specific cultural needs while delivering content on a basic and comprehensive level. It helps the target user from a social conservative background and in resource-poor circumstances to benefit from a digital educational solution.

## Introduction

Adolescent women in resource-poor and rural regions of Kenya between the ages of 15 and 25 years are most vulnerable and affected by the limited access to sexual health education and information due to a lack of financial resources, limited access to education, and a lack of infrastructure [1,2]. According to the Kenya Demographic and Health Survey of 2022 [3], 20% of girls in Kenya drop out of school during their menstruation. Young adults, aged 15-24 years, in sub-Saharan countries have greater unmet contraceptive needs than adults; on the contrary, these countries have the highest birth rates in the world [4]. Around 42% of the participating 1110 young women in Kenya stated that they did not want to become pregnant, but almost half of the participants never used contraception [5]. The consequences of nonexistent education not only are of a health nature (eg, dangerous abortions, lack of maturity of the body) but also affect the socioeconomic well-being of families where financial concerns already exist [6,7].

The 17 Sustainable Development Goals (SDGs) of the United Nations (UN), as a worldwide plan, seek to ensure peace and prosperity: SDG 3 seeks health for all people regardless of age, SDG 4 seeks sustainable and high-quality access to education, and SDG 5 seeks to “achieve gender equality and empower all women and girls to achieve self-determination” [8] and the willingness to ensure access to contraceptive information and sexual health knowledge [1].

Free accessibility and knowledge of sexual health care enable women and men to self-determine sustainable decision-making by taking responsibility of their own body and meeting global health standards, empowering themselves. Sexual health education is not represented in the national school curriculum of Kenya, and therefore, teachers are not trained accordingly to address this topic within classrooms [9]. Additionally, sexual health issues are addressed differently or concealed, differentiated by religion, tribe, and family and affiliation [10,11]. However, the taboo against contraceptive practices within society can have far-reaching consequences, such as fatal abortions to prevent unwanted pregnancies [7]. In slums and low-income regions, 50% of residents are aged 24 years or younger [12].

As solution strategies, digital technologies have a high potential, as Kenya has grown and developed into a pioneer of digitization in Africa, which also includes adolescent women from a low-income background who have access to digital media platforms [2,13].

In this scientific work, the concept and requirements of a future digital educational tool were developed to meet the challenges of limited access to knowledge, especially basic sexual knowledge. The aim of the web-based app is to provide free digital information about contraception and menstruation in order to empower and educate, as knowledge is power. Therefore, attention was paid to the accessibility of content, respecting the users’ cultural and religious situations and stigmatization. Since the target group has a different cultural background and the design process took place from a European cultural point of view, the user was incorporated into the process. As cultural differences influence product usability, Barber and Badre [14] summarized them as “culturability.” Culture is a kind of glass through which those involved perceive the world [15]. Therefore, this research falls under the term “cultural ergonomics,” developed by Smith-Jackson et al [16], where culture is embedded in the development process of designing human-computer interaction systems.

To identify needs and design principles, a reduced user-centered double diamond model approach was applied and expanded using cultural means based on Rau et al [17], Lachner et al [18], and Laws et al [19] (see Figure S1 in Multimedia Appendix 1), resulting in an intercultural research model (see the *Methods* section). The intercultural research model provides users from a specific culture with an option to use product developments of foreign cultures and increases accessibility and usability. It connects different cultural circles with different points of views. In the course of globalization, this is a necessary process [20].

## Methods

### Design: Double Diamond Model and Culture

Design thinking approaches enable creative freedom for addressing social and cultural issues, as well as serving and satisfying human needs. Exploring different perspectives on the issue of sexual health education in Kenya offers an advantage of additional learning about obstacles, in addition to the literature, and is essential for an innovative process. When facing challenges, according to Plattner et al [21], design thinking paves the way for innovative and necessary products to be developed. Nevertheless, design thinking requires collaboration with diverse working groups and an iterative improvement mechanism by focusing on the end user [22-24] and imprinting cultural ergonomics to identify the extent and limitations to which a product is usable and accessible [16]. Kroeber and Kluckhohn [23] highlighted the culture consistency of unconscious and conscious behavior, which is shared and transmitted within a social group. When identifying in the design thinking process, the target group’s needs and culture are of significant importance [21]. According to Barber and Badre [14], the decision-making process is determined by how people feel, behave, communicate, and think, as well as other behaviors and numerous characteristics [17,23].

Underlying the design thinking approach, the double diamond model is a static process that does not consider intercultural principles. Considering intercultural principles is based on the intercultural development process model of Rau et al [17]. The original intercultural development process model consists of 6 product development phases, with different document requirements and cross-cultural design studies (see Figure S2 in Multimedia Appendix 1). Phase 1, product development, identifies user requirements, who the user is, and what should be developed. Phase 2 defines the concept, creating a cross-cultural design philosophy to be adapted to the respective culture. Phase 3 drafts and specifies the product as a detailed requirements engineering analysis by including the respective culture and end user. Phase 4 onward, the model includes software development. Figure S3 in Multimedia Appendix 1 shows the newly constructed intercultural research model, aligning culture and the design thinking approach features from Rau et al [17], Lachner et al [18], and Laws et al [19] integrated into the problem room of the double diamond model of the Nielsen Norman Group. This offers the basis for cultural and user-centric design by systematically including cultural aspects in the development process and emphasizing on elaborate innovative solutions for complex problems.

In the discover phase, the user environment is analyzed based on in-depth literature research and semistructured interviews focusing on participants’ perspectives and personal needs to discover and highlight the problem from all perspectives. Problem-centered interviews focus on specific challenges, fundamentally expanded by personal perspectives.

Therefore, to enhance the research quality in the discover phase of our model, interview guidelines were defined and structured in alignment with the 32 COREQ (Consolidated Criteria for Reporting Qualitative Research; see Multimedia Appendix 2) [25,26]. This allowed spontaneous, freer interactions with the participants [27,28]. The COREQ guidelines recommend that pilot phase interviews be conducted to primarily define the problem and to ensure a basis for the subsequent qualitative content analysis [23] via expert interviews. We conducted semistructured interviews of 12 volunteers in the pilot phase. After analyzing and identifying the problem, we conducted 5 problem-centered, semistructured volunteer expert interviews, followed by qualitative content analysis based on audio-recorded data [26,29]. The interviews were divided into 6 sections: personal information, affiliation to tribes or religions, experience and knowledge of pregnancies, understanding of contraception, access to technology, and experience with digital health apps.

In the define phase, we defined the problem, focusing on the user and defining the environment, as well as differentiating the user from the customer, without neglecting cultural components by considering “cultural ergonomics,” a term created by Smith-Jackson et al [16]. This resulted in primary and secondary personas, an antipersona, and empathy maps and storyboards [17], as shown in Figures S3-S5 in Multimedia Appendix 1, which helped develop a resulting framework for requirements engineering analysis. According to Cooper et al [30], personas are identified to obtain a clear understanding of the target user (see Figures S8-S10 in Multimedia Appendix 1). The primary persona defines the target group, the secondary persona illustrates a user in alignment with the primary persona but with additional needs, and an antipersona demonstrates who will not benefit from the developed product due to differences [30]. Based on the identified personas, empathy maps were created for the user’s emotional understanding [31] (see Figures S11-S13 in Multimedia Appendix 1). It is important to appreciate the user’s context [31,32] by illustrating storyboards for a broader understanding (see Figures S14 and S15 in Multimedia Appendix 1). The illustration of storyboards simplifies the derivation of requirements in the preliminary stage. The discovery and define phases resulted in a requirements engineering analysis framework for which the categorization was based on 6 of the 7 interaction principles of the DIN EN ISO 9241-110 standard (where DIN refers to the Deutsches Institut für Normung, EN is European, and ISO is the International Organization for Standardization) [33]. The original sixth principle, “robustness against usage errors,” was excluded in this study and will be analyzed in later studies. Nevertheless, the current implementation of requirements already increased accessibility and usability to decrease usage errors. The solution room was divided into the develop and deliver phases based on the original Nielsen Norman Group’s double diamond model, which will be considered in later studies.

### Ethical Considerations

The study was conducted in accordance with the Declaration of Helsinki and approved by the Institutional Review Board (or Ethics Committee) of University Witten/Herdecke (protocol code S-119/2022, date of approval August 6, 2022). Signed informed consent for publication was obtained from all participants after providing them with a written information sheet and verbal explanation of the study context, the intended procedure, and data usage. All participants were given a copy of the signed informed consent as well as confirmation of anonymization of study data. The study data were anonymized, so participants cannot be identified. Participants did not receive any financial compensation for their participation.

### Recruiting and Participants

Due to data saturation, 5 expert interviews were conducted based on the study hypothesis [34] and the results of the 12 previously conducted pilot phase interviews to define the problem, in alignment with the principle of theoretical data saturation, after which new data provide no additional insights and the optimal sample size is reached [35]. For the sample size of 12, volunteer pilot phase participants were approached and recruited by local health care community workers from 2 community centers in Kenya based on the participants’ personal interest in and willingness to voluntarily contribute to the development of a digital sexual health education app. The 2 community centers are in the Langas slum around the city of Eldoret, Kenya. The Langas slum has around 300,000 inhabitants [36], reflecting the majority of Eldoret households.

To ensure meaningful data quality, participants, as representatives of the target user group, were limited to the female gender, aged between 15 and 25 years, and living in a low-income area with limited access to sexual health education.

For the 5 expert interviews, the focus was not the persona or the biography itself but, rather, the experience in the relevant context and expertise regarding the identified problem [37]. We interviewed 4 (80%) Kenyan and 1 (20%) US expert, all located in West and North Kenya, working in the field of women empowerment and interacting with the target user group. The expert interview participants were recruited and identified by the first author (CS) based on their local impact on sexual health and women empowerment and work with the target female user group aged between 15 and 25 years [28]. Sample characteristics of the interviewed participants are provided in Figure S4 in Multimedia Appendix 1.

### Data Analysis

The interviews were conducted by CS, who is female and the project initiator at the Fraunhofer Institute for Software and System Engineering (ISST) since 2021. All interviews were audio-recorded using Microsoft Teams and coded by authors CS and AR [26]. The 12 pilot phase interviews were transcribed and analyzed, laying a foundation for the required low-cost digital app focusing on menstruation and contraception. This was followed by the 5 expert interviews to deepen the research and to represent the respective research area [37], with a duration of 30-70 minutes, in accordance with COREQ guidelines [26]. Nonwillingness to participate was not detected during the interviews [26]. Guaranteeing data consistency and for data coding, all interviews were paraphrased when transcribing for qualitative research [26], as identified by Meuser and Nagel [37], while using the affinity mapping method [38]. To do so, expert interview results were noted on 401 different-colored Post-it notes, one color for each anonymized expert, identified as B1-B5 (see Figure S5 in Multimedia Appendix 1) [26,38]. Collecting B1-B5 responses on Post-it notes enabled categorization into different topic clusters (see Figures S6 and S7 in Multimedia Appendix 1). We also created personas, empathy maps, and user storyboards to illustrate requirements and design recommendations, as described by Laws et al [19].

## Results

### Expert Details

This work followed user- and human-centered design principles to define the requirements for developing a sexual health education app. To identify the problem, 12 pilot phase interviews were conducted. Due to low-quality responses, analysis was limited. In addition, due to language barriers and neglected audio quality, as well as background noises, we could not reach a common understanding of the interview responses. Based on a few usable responses from the pilot phase participants, the necessity of a digital app focusing on sexual health education was clearly presented, but no interest in a pregnancy-tracking app was identified. Based on the 12 pilot phase interviews, we conducted high-quality expert interviews (n=5) to deepen the research and collected the expert responses on 401 Post-it notes of different colors, one color for each expert, defined as the affinity mapping method [38] (see Figures S5 and S6 in Multimedia Appendix 1). The 401 Post-it notes were categorized into 8 different topic clusters, (see Figure S7 in Multimedia Appendix 1) and built a basis of the 3 personas, 3 empathy maps, and 2 storyboards, leading to the requirements engineering analysis.

#### Cluster 1: Person

This cluster included information about the participating experts. Different perspectives were highlighted, and the women’s background was of particular importance. The first participant, B1 (yellow), a Kenyan social worker, originally from the rural village of Kisumu, works with women at a nongovernmental organization (NGO). She represented a rather conservative view, in which the values of religion and culture appear to be important. B2 (blue) is American and represents a Western point of view but is familiar with the challenges of the Kenyan population. Due to her experience in Kenya, India, and Guyana, she could contribute expertise. The third participant, B3 (orange), is from the rural region of Turkana in Kenya. In addition to her involvement in women empowerment groups, she is the mother of an unplanned, now 9-year-old son, for which she was abandoned by her family. She has the perspective of someone with a prior lack of sexual health education based on personal experience. B4 (purple) is a Kenyan student at Moi University in urban Eldoret. B4 contributed her perspective as an educated student from urban Kenya, where women are more likely to have access to education [39]. B5 (green) studied at the university in Eldoret and is originally from the coastal city of Mombasa. B5 has founded her own NGO focusing on sexual and reproductive health and rights (SRHR) and gender-based violence. She is familiar with different perspectives from rural Kenya and the Western perspective as she has grown up in Kenya, Germany, and the United States, enabling her to impart knowledge, especially in the field of social belonging in Kenya. Cluster 1 showed that each expert had a different social affiliation and provided perspectives on the research, offering a multiperspective base for the requirements engineering analysis.

#### Cluster 2: Social Belonging

This cluster was categorized into *tribal community*, *religion*, and *gender roles*. According to B4, tribal communities are trying to maintain their cultural habits, whereby education for women does not have a high priority. According to B1, who is a member of the Luhya tribe, the communities frequently meet to exchange and socialize. Religion has a high priority. B1 stated that the majority are Christian, and in the coastal area, the majority follow Islam. B5 believes that religion is of great importance, especially in low-income areas, as it provides direction. She stated that, frequently, religion is followed over government rules and law. B5 also stated that traditional gender roles are deeply rooted, and frequently, money is paid in the case of sexual abuse and rape.

That means when young ladies go through things like rape, people don’t report it and people don’t talk about it. It’s handled in the community sense.

B5

#### Cluster 3: Education

Cluster 3 was divided into *schooling system*, *reasons to drop out of school*, and *sexual education*. Most children aged 4 years go to school, as it is free. Secondary education is fee based, where primarily boys go to secondary school compared to girls, states B2. B5 stated that it is unusual in some parts of Kenya for women to go to secondary school. Reasons for school dropouts are diverse. B1 and B4 stated that early pregnancy is a reason, as well as the long walking distance to school facilities, according to B5. Sexual education as well as contraceptive education are almost nonexistent in secondary school, as stated by B1, B3, and B5. B2 argued that the limited education is due to the government and a conservative mindset:

The ABC—abstinence, be faithful, condoms—are being taught in that order.

B2; Figure S7, Cluster 2, Multimedia Appendix 1

B2 also stated that NGOs endeavor to provide contraceptive, family planning, and sexual health education.

#### Cluster 4: Marriage

Although marriage under 18 years is not legally permitted, early marriage continues to take place, especially in conservative, impoverished families, according to B5. B3 stated that young people in Turkana get married from the age of 12 years. B4 specified an age gap of 15-17 years for the region around the city of Eldoret and the Langas slum. B2 mentioned the rescue of a 9-year-old girl from marriage by her NGO. The girl was being married off to the village oldest by her parents for money. B5 explained that the girl ran to the NGO with bleeding genitals. B5 also explained that girls and families hope to escape difficult situations, especially not having enough food, through marriage. There is hope, but most of the time, the girl remains impoverished. Traditional tribes, such as the Maasai, continue to practice early marriage, whereas other tribal communities strictly oppose it, such as B5’s tribe. B2 added that the early marriage construct is slowly changing, also in rural areas. When the government becomes aware of such cases, it breaks off the marriage. However, the cohesion within the community makes it difficult.

#### Cluster 5: Pregnancy

Kenya has one of the highest birth rates among teenagers [3]. B3, who got pregnant at 14 years, emphasized the unplanned pregnancy aspect. B1 said that the average age of pregnancy is 13-14 years. B4 stated:

For me, most girls don’t get pregnant because they want to get pregnant. They get pregnant because they lack…they lack the knowledge, they’re not told if you engage in unprotected sex, you’re going to get pregnant.

B4; Figure S7, Cluster 5, Multimedia Appendix 1

However, according to B4, the stigmatization and taboo around sexual interactions harbor danger, as women face an increased risk of mortality and struggle with psychological problems. Expecting mothers frequently realize their pregnancy at a late stage. B3 learned in month 5 of her pregnancy, due to stomach cramps, during a hospital examination. She stated that after giving birth, she did not receive any assistance and was left to her own devices. In many communities, unplanned pregnancies go hand in hand with marriage:

So, because you’re pregnant, you get married.

B4; Figure S7, Cluster 5, Multimedia Appendix 1

In other cultures, however, girls are dispossessed. They lose respect in their family and neighborhood and must leave them, as did B5 (see Figure S7, Cluster 5, in Multimedia Appendix 1). She criticized that most women to not get an opportunity to know better—the fear of an unplanned pregnancy without being married is bigger than the fear of getting infected with HIV. B4 and B5 summarized the necessity of sexual education for young girls to make informed and conscious decisions concerning sexual interactions.

#### Cluster 6: Contraception

Cluster 6 included *accessibility*, *knowledge of contraception*, *false information*, *recognition of* *contraception*, *taboo*, and *sexually transmitted diseases* (STDs). B3, B4, and B5 said that accessibility is impaired by religion, social affiliation, tribal communities, and location, as well as a lack of information. B1, with a traditional perspective, prioritized abstinence and questioned whether women should be informed about hormonal contraception. B3, B4, and B5 emphasized the lack of knowledge and the necessity to educate, as no one can profit from contraception if no one knows about it. Additionally, they stated that condoms, pills, and injections are most frequently used as contraceptives, but criticized the distance to health care centers. On the contrary, according to B5, most drugs are being diluted and mixed, as most medicine sold is not registered by the government. It is of great importance that contraceptives be approved and certified by the health ministry. According to B3, women who have not yet given birth do not receive contraceptives from organizations.

And in some hospitals, they won’t even allow if you’ve not given birth…like for me, you’ll just have to use the pills or over-the-counter drugs. You don’t have these other implants to help protect you from pregnancy. Yeah, they forbid it, some hospitals.

B3; Figure S7, Cluster 6, Multimedia Appendix 1

The category *knowledge of contraceptives* highlights the lack of sexual education in schools. According to B3, in older generations, woman usually have up to 10 children but do not know about sexual education.

A sister of mine, who’s younger than me, but she already has, like, 4 kids, because she got married at a very, very young age and she didn’t know how to protect herself.

B3; Figure S7, Cluster 6, Multimedia Appendix 1

Most teenagers are told to practice abstinence. According to B3 and B5, knowledge is a need, as not all girls have an elder sister to teach them; they have only brothers and fathers with traditional points of view. B5 stated taboo and stigmatization are leading to deadly illegal and unsafe abortions. The category *recognition of contraceptive methods* illustrates, on the one hand, the presence of cultural and religious stigmatization in tribal communities, according to B1; especially, the elderly call it the “Western impact” and do not agree on it, according to B4. On the other hand, B3 stated that marriage is of great importance, as a woman’s task is to birth children. In addition, B5 mentioned that information found on the internet is considered false information.

If we promote contraceptives, then we are allowing something that is not [culturally] accepted.

B1

Religion has great importance, abstinence is being taught, and sexual activity before marriage is taboo. Therefore, according to B1, contraceptives are only for women who already have birthed a child. In addition, B3 mentioned the *male perspective*, which is mainly negative. Partly, women need approval of their husbands for contraceptives. B4 and B5 mentioned the *parent perspective* and stated that parents need to be educated as well. B4 mentioned especially that *young women* should be educated from the age of 15-25 years, as they are interested and willing to understand the different methods oriented on Western culture. According to B4, a lot of *false information* around contraceptives exists. B2, B3, and B4 stated that the success of a digital app would be in decreasing false information. According to B1, B3, and B5, sexual education is mostly *taboo*. Most parents forbid contraceptives but do not provide an explanation as to why.

We don’t talk about contraceptive methods with [our] parents.

B5; Figure S7, Cluster 6, Multimedia Appendix 1

In addition, *STDs* were addressed. B2 and B5 mentioned that the benefits of contraceptive education will not only have a positive impact on unplanned pregnancies but also decrease STDs. According to B4, around 100 women test positive for HIV per week. Additionally, B2 stated a discrepancy between contraceptives and STDs:

Do they also [equate it to] not having babies, or how much do they know?

B2; Figure S7, Cluster 6, Multimedia Appendix 1

#### Cluster 7: Technology Aspects

B5 distinguished between 2 groups, one with access to the internet in rural areas and one without. B1 estimated that around 20% have access to the internet and smartphones. B4 estimated that every 3 girls out of 10 have a smartphone in Kenya. All participants agreed on the increasing percentage of smartphone usage. According to B1, if not a smartphone, the girls have a mobile phone, with which they use the Kenyan online payment system M-PESA (“M” for “mobile” and “Pesa” for “money” in Swahili) from the local telecommunication provider Safaricom. B1 and B2 stated that internet access in rural areas is expensive, but NGOs in rural low-income areas provide access to it. Additionally, Starlink will be provided in Kenya from 2024 onward. B3 stated:

But through the organization (NGO) coming there, it’s like…it’s made it grow, and more people can access the internet here through their phones just sitting at home…Like for me, I raised my son through Google.

B3; Figure S7, Cluster 7, Multimedia Appendix 1

B5 stated that at least 1 person in a teenage friend group has a smartphone, and additional computer rooms are available in schools, community centers, and NGOs. B5 mentioned the limitation of accessibility and using the app due to sensible and intimate content.

#### Cluster 8: Application

Cluster 8 focused on the opinions and ideas concerning a digital sexual health education app, categorized into *recognition*, *proposal*, and *interaction*. Under *recognition*, B3, B4, and B5 showed strong support for a digital educational app. Not everything has to be included from the beginning, but it is a starting point and an opportunity to learn. B3 said that technology can encourage girls to get educated. B5 emphasized the option to get education via a digital app:

It’s like a big sister you never had. You want to know more information about sexual health? This is the right place for you to go. What do you want to know about it?

B5; Figure S7, Cluster 8, Multimedia Appendix 1

B5 and B2 gave a *proposal* for optimal usage and the importance of access to information, considering the language barrier and increasing attention to digital apps. An *interaction proposal* was raised to implement creative audio files and visible options. Graphics and videos increase accessibility and usage, as stated by B2 and B3. According to B2, B3, and B5, also important is a comic and creative illustration. Swahili and English should be included, and for those without access to the internet, an offline version should be made available.

### Data Analysis Leading to a Requirements Engineering Analysis Framework

Our culturally sensitive user-centered design approach required analyzing the responses obtained in pilot phase (n=12) and expert (n=5) interviews to fully understand the needs of users from a foreign culture. The first phase of the intercultural research model, discover, was concluded by dividing the responses into 8 different clusters, followed by the next phase, define, identifying 2 personas and 1 antipersona, creating 3 empathy maps, and creating 2 storyboards for the intercultural research model (see Figures S8-S15 in Multimedia Appendix 1). Personas 1 and 2 (see Figures S8 and S9 in Multimedia Appendix 1), named Ivy and Naomi, showed the personal needs of the user in comparison to the antipersona, Zawadi. The empathy maps (see Figures S11-S13 in Multimedia Appendix 1) highlighted the emotional needs of the user [31] in correlation with the sensitive topic of sexual health education. Requirements were highlighted for a digital app, which must be considered when creating an intimate and trustworthy app. Empathy boards built a basis for the storyboards, which simplified the derivation of the requirements. The storyboards visualized the results (see Figures S14 and S15 in Multimedia Appendix 1). The variables language, religion, and culture impact sexual health education. The limitations of the prototype, such as language barriers, necessitated its simplification for users’ understanding of the storyboards. The storyboard for the primary persona, Ivy, representing the target group, focused on a potential use case of being informed by sexual health education and learning about a sexual health education app through a flyer. The visualization simplified the derivation of requirements as a result of requirements engineering analysis. Each specific requirement, as described in Tables 1-4, was assigned an ID, a type (functional [FA] vs nonfunctional [NF]), and a prioritization (marked with “X” if considered for the low-fidelity prototype). The results increased the accessibility and usability of the target group and counteracted misleading conclusions.

**Table 1:** Requirements engineering analysis for the digital sexual health education app in a resource-poor region and categorization based on the DIN EN ISO 9241-110^a^ standard for task appropriateness^b^.

ID	Type	Requirement	Priority
TA1^c^	NF^d^	The app has a purely informational character, without consciously influencing users.	—^e^
TA2	NF	All the content of the app is available offline after downloading.	—
TA3	NF	The app can be accessed free of charge at any time.	—
TA4	NF	The contents of the menu item “Contraceptive methods” are provided and checked by health professionals; they provide scientifically well-founded information. The indication that the information is checked by specialists appears under the textboxes.	—
TA5	FA^f^	Under the menu item “Contraceptive methods,” for each form of contraception, an introductory text with a short description is shown, and it is explicitly mentioned that contraceptive methods prevent pregnancy when used correctly.	—
TA6	FA	Under the menu item “Contraceptive methods,” category “Condoms,” the introduction describes that condoms can be used to prevent pregnancies as well as prevent venereal diseases.	—
TA7	FA	Under the menu item “Contraceptive methods,” for each contraceptive method, a textbox appears with information about how to apply each method.	—
TA8	FA	Under the menu item “Contraceptive methods,” a textbox “Myths and facts” appears for every form of birth control to eliminate the misconceptions of the users.	—
TA9	FA	Under the menu item “Contraceptive methods,” a textbox appears with information concerning security for every form of contraception.	—
TA10	FA	Under the menu item “Contraceptive methods,” an information box with price indications appears for each form of contraception.	—
TA11	FA	Under the menu item “Contraceptive methods,” advantages and disadvantages of each form of contraception are listed.	—
TA12	FA	Under the menu item “Contraceptive methods,” an impact score of each contraception method is shown.	X^g^
TA13	FA	Under the menu item “Contraceptive methods,” category “Pill,” an area appears that lists the different providers and limited side effects when using different pill brands.	—
TA14	FA	Under the menu item “Contraceptive methods,” for each contraceptive method, an indication appears that in health care facilities, more detailed information can be provided to the user, as well as a hint that the app cannot replace a specialist. There is also in the menu an item called “Health care facilities,” providing the locations of different facilities.	X
TA15	FA	Under the menu item “Health care facilities,” the users can check whether condoms are free of charge and available in the respective facility.	X

^a^DIN: Deutsches Institut für Normung; EN: European; ISO: International Organization for Standardization.

^b^“An interactive system is task-appropriate if it supports users in completing their tasks, that is, when the operating functions and the user-system interactions are based on the notable properties of the task” [33]. In an intercultural context, the expectations of the users should be met and lead them to their goal [31]. Information that is not suitable for the users can be stressful [33]. Accordingly, the app is intended to support users in dealing with such a sensitive topic by reducing uncertainties and answering questions.

^c^TA: task-appropriate.

^d^NF: nonfunctional.

^e^Not applicable.

^f^FA: functional.

^g^X: applicable.

**Table 2:** Requirements engineering analysis for the digital sexual health education app in a resource-poor region and categorization based on the DIN EN ISO 9241-110^a^ standard.

Requirement and ID			Type		Requirement		Priority
**Self-descriptiveness^b^**							
	SD1^c^	NF^d^		Mental models (eg, the hamburger icon) are being used within the app.		—^e^	
**Controllability^f^**							
	C1^g^	FA^h^		A back arrow can be used and the logo icon and the hamburger icon used to navigate through the app.		X^i^	
	C2	FA		The menu item “Contraceptive methods” is further divided into subcategories, from which users can reach each method (eg, pill->hormone-based pill and emergency pill).		X	
	C3	FA		As part of the app, a menu item called “Health care facilities” is present. It shows health facilities surrounding the users, depending on the location.		X	

^a^DIN: Deutsches Institut für Normung; EN: European; ISO: International Organization for Standardization.

^b^“Wherever required by the user, the interactive system provides appropriate information that make the capabilities of the system and its use immediately apparent, without that this requires unnecessary user-system interactions” [33]. In an intercultural context, the app should be always clear and understandable to navigate [31]. Due to the limitation of the target group, a certain affinity toward technology can be assumed. It is important to have well-known mental models so that users can recognize them in the app.

^c^SD: self-descriptiveness.

^d^NF: nonfunctional.

^e^Not applicable.

^f^“The interactive system allows the user to take control of the user interface and retain the interactions, including speed, sequence, and customization of user-system interaction” [33]. In an intercultural context, due to the exploratory character, it is essential for the user to control the app according to their needs. This means, for example, that the user can always go to the previous page [31].

^g^C: controllability.

^h^FA: functional.

^i^X: applicable.

**Table 3:** Requirements engineering analysis for the digital sexual health education app in a resource-poor region and categorization based on the DIN EN ISO 9241-110^a^ standard for conformity to expectations^b^.

ID	Type	Requirement	Priority
CE1^c^	NF^d^	Technical terms are explained to the users in simplified form.	X^e^
CE2	NF	The app is supported in ​​English and Swahili.	X
CE3	NF	All text passages can be read out loud through a specific function provided (speaker symbol).	X
CE4	NF	In the app, text inputs are avoided due to knowledge barriers. Necessary text entries (eg, when entering one’s birthday) are possible via a voice function (verbal support).	X
CE5	NF	Every menu item, birth control method, and headline is illustrated in a sketch-like representation, in addition to the text (visual support).	X
CE6	FA^f^	The logos of the participating organizations or NGOs^g^ are included with a link to the organizational webpage.	X
CE7	FA	As part of the app, a menu item “Organizations” is listed, which directs users to Kenyan social media platforms related to sexual health.	—^h^

^a^DIN: Deutsches Institut für Normung; EN: European; ISO: International Organization for Standardization.

^b^“The behavior of the interactive system is predictable based on the context of use and generally recognized conventions in this context” [33]. In an intercultural context, even if the app confronts users with a taboo topic, they should not feel overwhelmed. No menu item should contain any surprising information, and every menu item should correspond to the users’ existing knowledge and experience [31].

^c^CE: conformity to expectations.

^d^NF: nonfunctional.

^e^X: applicable.

^f^FA: functional.

^g^NGO: nongovernmental organization.

^h^Not applicable.

**Table 4:** Requirements engineering analysis for the digital sexual health education app in a resource-poor region and categorization based on the DIN EN ISO 9241-110^a^ standard.

Requirement and ID			Type		Requirement		Priority
**Learnability^b^**							
	L1^c^	FA^d^		The users get an introduction when registering, during which the user learns about the content and the different functions of the app. The introduction is informed, and the users is advised to take a quiet and undisturbed moment to read the content.		X^e^	
**User retention^f^**							
	UR1^g^	NF^h^		Within the app no religious, traditional, and tribal affiliations are being expressed.		—^i^	
	UR2	NF		A personal atmosphere is created by using pronouns like “I” and “you” are used.		—	
	UR3	NF		The app can be used without logging in. This allows the users a feeling of safe place, without the feeling of judgement and exploration.		X	
	UR4	FA		In the introduction, a fictional character appears (Linda and Leo) which are used as metaphor as the big sister or brother.		X	
	UR5	FA		The logos appear on the start page of the app organizations or NGOs^j^ involved in the market launch, whereby the social impact character is emphasized.		X	
	UR6	FA		Several African women appear on the screen at the homepage of the app to enable a sense of connection.		X	

^a^DIN: Deutsches Institut für Normung; EN: European; ISO: International Organization for Standardization.

^b^“The interactive system supports the discovery of the abilities and their use, it allows the interactive system to be explored (‘tried out’), minimizes the learning effort and offers support when learning is required” [33]. In an intercultural context, since the users may have a different know-how of how to use a mobile app, they should be supported in how to use it so that they can focus on the content [31].

^c^L: learnability.

^d^FA: functional.

^e^X: applicable.

^f^“The interactive system presents functions and information in an inviting and motivating manner, and thus promotes continuous interaction with the system” [33]. In an intercultural context, the system should create trust [33] and thus promote the metaphor of a safe place.

^g^UR: user retention.

^h^NF: nonfunctional.

^i^Not applicable.

^g^NGO: nongovernmental organization.

## Discussion

### Principal Findings

The results of the interview analysis in the study prove the usability and necessity of free, accessible educational sexual health content, while respecting cultural and religious differences when developing a digital app [14]. The study and its concluding requirements engineering analysis (see Tables 1-4) determined barriers to using a digital sexual health education app, such as a conservative cultural background, classic text communication, and the influence of social affiliation within society, which should be enhanced through visual and auditory channels as well as a fictional character in the app. One reason for a lack of knowledge is the stigmas within society, such as partly resisting contraceptives, as the people are not sufficiently informed [10], and these stigmas are differently addressed based on religious and cultural backgrounds [10,11]. Misconceptions about fertility prevail, and side effects of contraception do exist [40]; however, acceptance depends on various factors, and improving sexual health is identified as a major necessity [41]. The requirements engineering analysis verified the need for a newly developed intercultural research model to ease the product development for foreign cultures and increase usability at a later stage. Additionally, in resource-poor regions, educational content needs to be addressed as different knowledge levels coexist, while granting universal free, accessible sexual health care education.

Kenya was used as a case study because a network of community centers, mobile infrastructure, and low-income regions for evaluation exist. Additionally, there is a lack of sexual education and knowledge of methods to prevent pregnancy and STDs, especially among young girls [1,4,9]. Digital technologies are becoming increasingly more available in resource-poor regions, as well as in rural areas of Kenya [42,43]. Kenya is known for the innovative and mobile low-cost payment app M-PESA [44], with which mobile penetration has increased to more than 78% [43]. It is proven that mass media reaches and educates young adults about sexual health [45]. Olamijuwon and Odimegwu [41] examined the dissemination of information about sexual health in Kenyan society and the acceptance of dissemination of educational content via digital tools with 936 young people in Kenya, Nigeria, and South Africa. They found that 84% of young people indicated digital media as suitable for communicating about sex education. Another study confirmed that adolescents in Kenya feel the need for easily accessible and trustworthy information about maintaining sexual health [46]. The UN has defined 17 SDG targets to eliminate limited access to sexual health education [8,47]. A digital app developed based on the identified requirements would target SDGs 3, 4, and 5 and increase universal access to sexual and reproductive health.

The well-established double diamond model approach, as a design thinking approach embedded with intercultural perspectives, created an intercultural research model for this study, especially focusing on cultural ergonomics [16], considering the design recommendations aligned with Laws et al [17], Rau et al [18], and Lachner et al [19]. Culture affects not only the development process but also the communication and management of design processes. Integrating cultural aspects helps meet user needs and understand different perspectives [16,21], thereby establishing empathy with the user. Establishing an intercultural research model proved useful for the given environment of the target group. Structuring qualitative, semistructured, and problem-centered interviews proved positive, as the structure could be adapted to each participant, and demonstrates the need for and potential of the app. A positive response to digital distribution was expressed, if other factors, such as social values ​​and detailed explanations, were considered. The 5 expert participants only interacted with the target user group but were not part of it. The results proved that a survey of 5 experts is sufficient to accumulate information. However, with fewer people, it would have been difficult in an intercultural context, since the understanding of each individual point of view expands the topic. The affinity mapping method was suitable for organizing and summarizing a wide range of information systematically [38]. The target group consisted of diverse people with different lifestyles and circumstances, which may have made finding solutions difficult. The target group had to be narrowed down further due to the excessive dependence of Kenyan women on their environment, particularly on their social affiliation with conservative circumstances, as shown by Ontiri et al [10] and Goodman et al [11]. In addition, the results demonstrated young Kenyan mothers interfacing within the primary target group, which indicates that the app will target not only young women before their first pregnancy but others as well.

In the future, this project must evaluate whether representations are clear and influence accessibility as well as to what extent the app, within certain regions, is accepted and whether differences can be identified. Results cannot be automatically transferred to every region. Nevertheless, the requirements engineering analysis, demonstrated in Tables 1-4, offers an orientation framework, as the design pays attention to different affiliations, which results in no disadvantage or differences.

For implementation purposes, first, a lean target group having access to a smartphone and interest in their own sexual health should be addressed. Both the interviews and the literature show that digitization is a growing process and so is the need for sexual health education. The app has great potential, given the safe and intimate surrounding created with the fictitious character in the app—“like a big sister” according to B5. As also shown by Green et al [46], a trusting atmosphere must be created in digital educational tools.

The identified requirements should be addressed when developing digital educational tools. In addition, the need for sexual health education should be clarified, especially regarding contraception for young women. In the future, for a prototypical elaboration, several women must be integrated into the evaluation—at least 5 women are recommended in order to increase scalability. In addition, prototype testing should be carried out with a specific target user group as this will determine accessibility and indicate the potential limitations and unpredictability of successful acceptability by users. It is important to question whether the model can be transferred to other cultural circumstances; this will be reviewed in future studies when wanting to generalize to create a broader impact on society and empower people via education.

### Limitations

Although the 12 pilot phase interview participants were part of the target user group, the results of the interviews were of low quality. The low quality was due to the addressing of intimate topics that are a taboo within society and can quickly lead to feelings of shame. Another reason for the low quality was language barriers, which made detailed statements difficult. In addition, the interview group might represent a certain bias; nevertheless, the interviews testified the necessity of a digital solution. Furthermore, women from conservative surroundings and living in poor circumstances cannot benefit from the app.

### Conclusion

This study identified and established a reusable framework in which taboo and intimate topics can be delivered without the stigmas of religion and culture in a basic and comprehensive way via a digital app to resource-poor regions. For this purpose, we extended the user-centered design thinking process of the double diamond model by integrating cultural perspectives [17], resulting in a specially developed intercultural research model. This scientific work integrated culture-specific aspects in the design process and provides an impetus to delivering accessible sexual health education in digital and mobile form to young women in low-income and low-resource regions, having special needs [1-3], at low cost and aligned with cultural ergonomics. We targeted women aged between 15 and 25 years in the resource-poor regions of Kenya, identified by the established personas. Adolescents’ lack of access to information about sexual health can have far-reaching consequences, especially for women [1]. The increasing digitalization in Kenya offers great potential; therefore, Kenya was used as a case study. The digital mobile-based educational app can provide accessible, free information, with a focus on education of contraception and menstruation.

In retrospect, it becomes clear that the app helps understand the challenges of users from a foreign culture. In particular, the contact within the target culture provides detailed opinions, far removed from the literature, and helps overcome hurdles.

Through the interviews, it became evident that a digital app is a suitable approach to obtain useful educational information about sexual health. The findings of the requirements engineering analysis indicate that classic text communication can be a barrier; therefore, it should be enhanced through visual and auditory channels. Audio files for the read-out-loud function should be added in Swahili and English to overcome the language barrier. Additionally, a fictional character should be created to lead the user through the app to minimize the feeling of shame and taboo by creating trust and intimacy, as a fictional big sister. The strong influence of social affiliations within society was evident during expert interviews. This can be due to the strong religious and cultural stigmatization around sexual health education. It is advised that an onside evaluation of the prototype be conducted with the specific target user group of 15-25-year-old women as the resulting solution requires a new evaluation. Likewise, expansion through an interactive chat with a fictitious big sister should be evaluated.

Expansion to other low-income regions should be considered, orienting along the framework and carried out using the established intercultural research model. Integration of the opposite sex in the research should be considered in the long term to create a holistic approach that provides access to improved sexual health in alignment with UN targets and universal standards. The overall use of online education tools focusing on intimate topics is correlated with accessibility and with understanding specific cultural needs, while delivering content on a basic and comprehensive level.

## Appendix

Multimedia Appendix 1 All personas, empathy maps, storyboards, and cluster categories for a good understanding of the groundwork needed to establish the framework of a requirements engineering analysis.

Multimedia Appendix 2 Consolidated Criteria for Reporting Qualitative Research (COREQ) checklist.

## Abbreviations

COREQ: Consolidated Criteria for Reporting Qualitative Research
DIN: Deutsches Institut für Normung
EN: European
FA: functional
ISO: International Organization for Standardization
NF: nonfunctional
NGO: nongovernmental organization
SDG: Sustainable Development Goal
STD: sexually transmitted disease
UN: United Nations

## Data Availability

All data generated and analyzed during this study are included in this published paper and Multimedia Appendix 1.
