# Supplementary material for: Creating an Intercultural User-Centric Design for a Digital Sexual Health Education App for Young Women in Resource-Poor Regions of Kenya: Qualitative Self-Extended Double Diamond Model for Requirements Engineering Analysis
Source: JMIR Form Res. 2023 Nov 3;7:e50304. doi: 10.2196/50304 (PMC10656664; doi:10.2196/50304)
Supplement: Multimedia Appendix 1 [file formative_v7i1e50304_app1.docx]

# Multimedia Appendix

*Figure S1: User-centered Double Diamond approach*


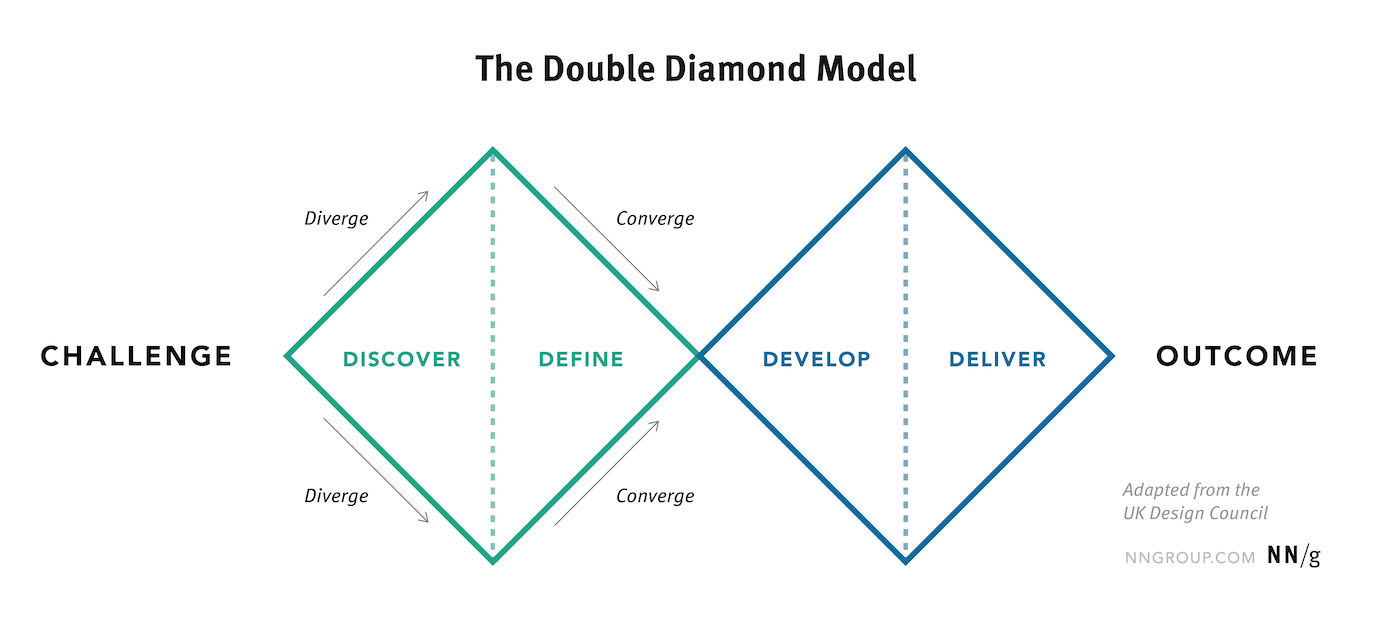


Source: UK Design Council, Nielsen Norman Group


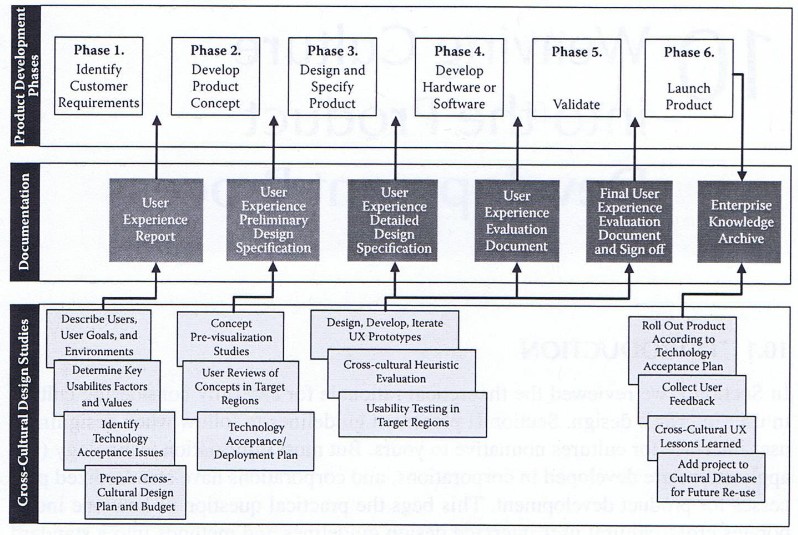
  *Figure S2: Intercultural Design during development process based on Rau et.al (2013)*

### Source: Intercultural Developmentprocess (Rau, Plocher & Choong, 2013, S.148)

Figure S3: Intercultural Research Model, own illustrated based on the Nielsen Norman Group Double Diamond Model and Rau et.al (2013)

**Problem**

***Discover***

***Define***

**Requirement definition**

**Problem Room**

Problem Statement

Interviews

***Requirement Engineering Analysis***

Storyboard

Persona

Empathy Maps

**Result**

### Source: own illustration

*Figure S4: Color coordination and characteristic overview of expert interview participants*

| Sample Characteristic Pilot Phase Participants | |
| --- | --- |
| Age | < 15 |
| Location | Rural Areas in Kenya, Slums |
| Occupation | Non – depending on community centers |

| Sample Characteristic Expert Participants | |
| --- | --- |
| Age | < 20 |
| Location | Nairobi, Eldoret, Lodwar |
| Occupation | Community Health Worker, Teacher, NGO worker |

Source: own illustration

*Figure S5: Color coordination of expert interview participants*

Source: Miroboard, own illustration

*Figure S6: Overview: Concluding of all ideas from the interviews of each participant*


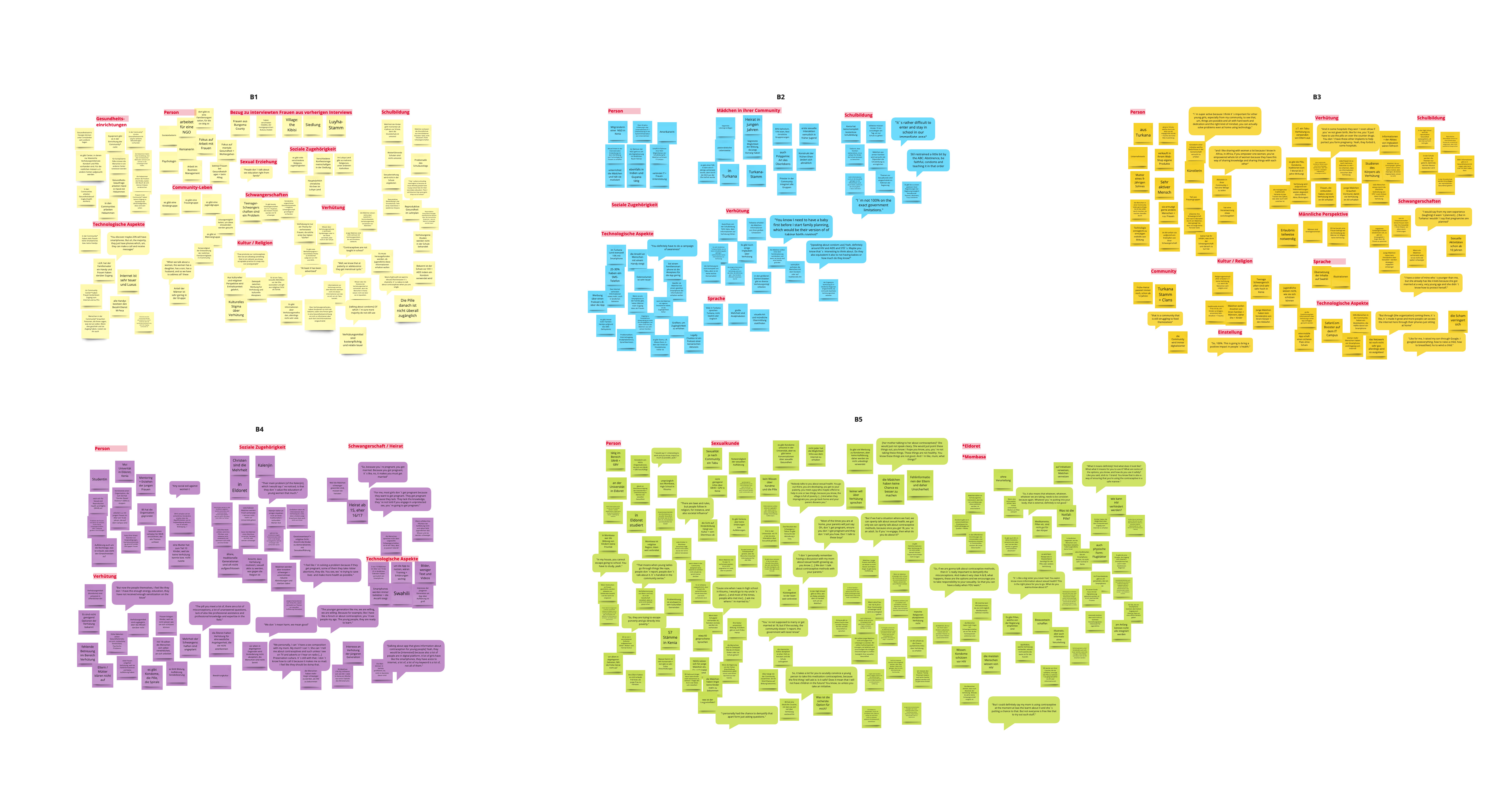


Source: Miroboard, own illustration

*Figure S7: Cluster Overview with coordinated colors of Expert Interview Participants*

*Cluster 1: Person*


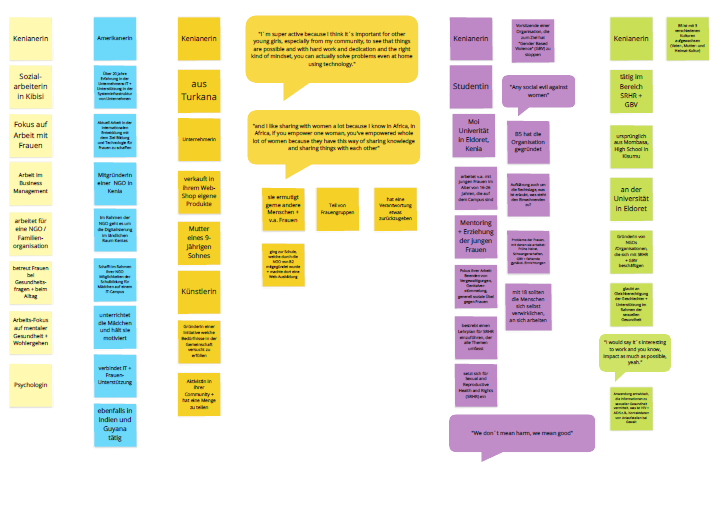


Source: Miroboard, own illustration

*Cluster 2: Social belonging*

Tribal Communities Religion Gender Roles


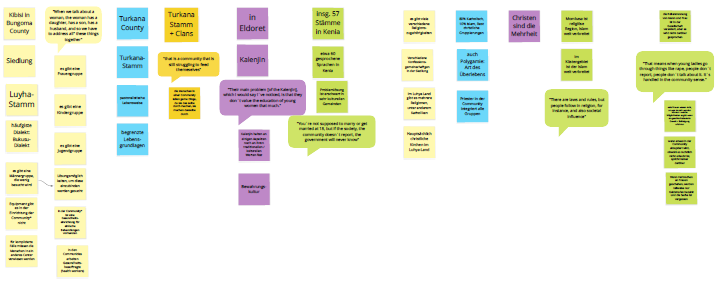


Source: Miroboard, own illustration

*Cluster 3: Education*

School System School Dropout Sexual Education


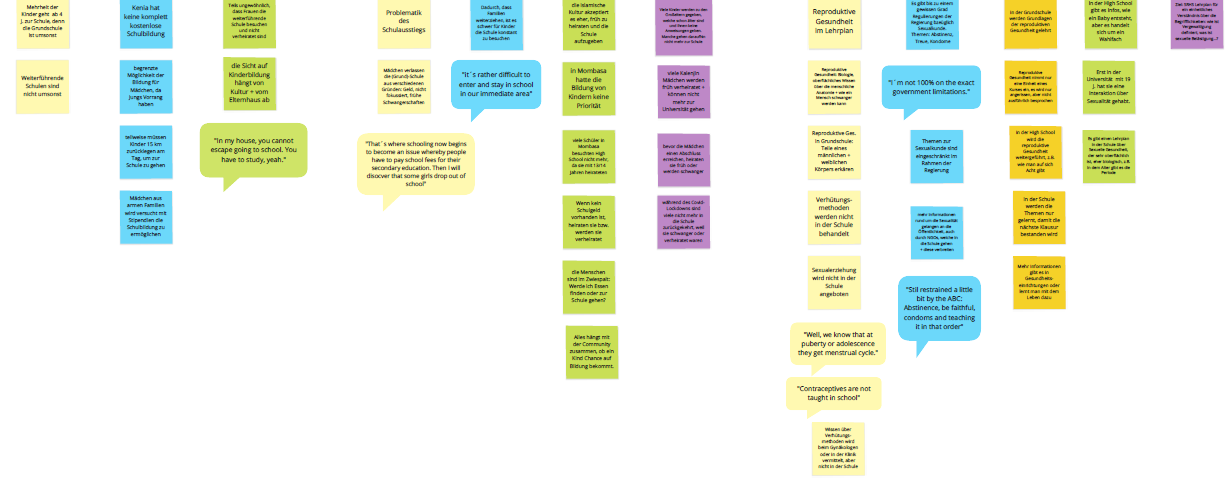


Source: Miroboard, own illustration

*Cluster 4: Marriage*


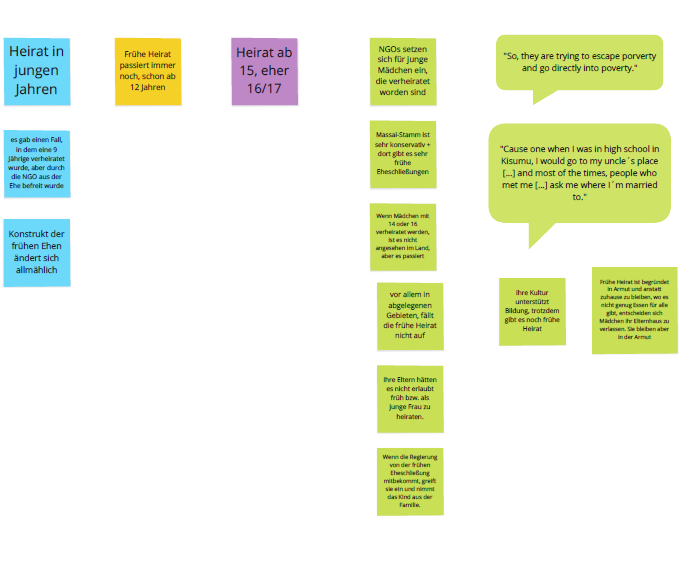


Source: Miroboard, own illustration

*Cluster 5: Pregnancy*


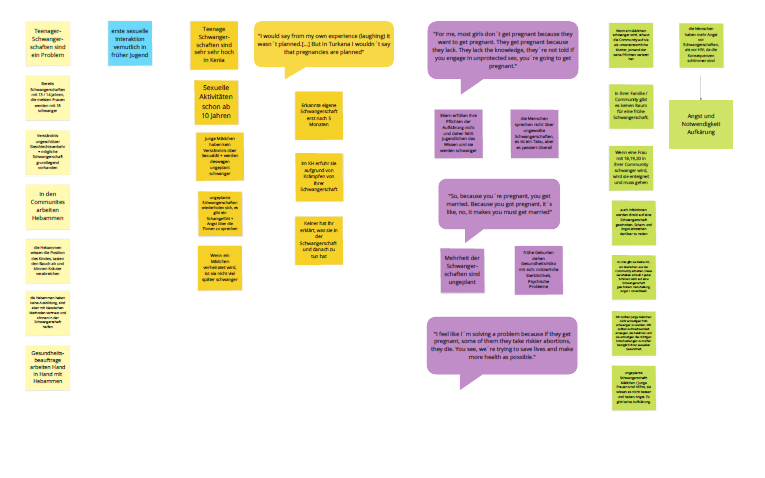


Source: Miroboard, own illustration

*Cluster 6: Contraception*

Accessibility Knowledge of contraception


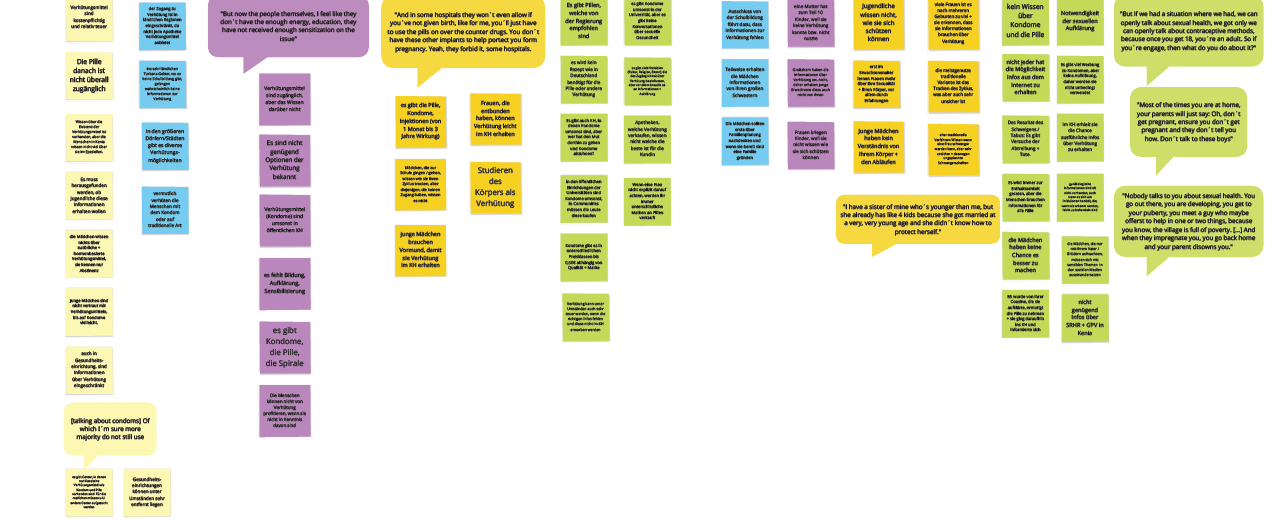


Recognition of contraception

Male Perspective Triable Communities Religion Parents


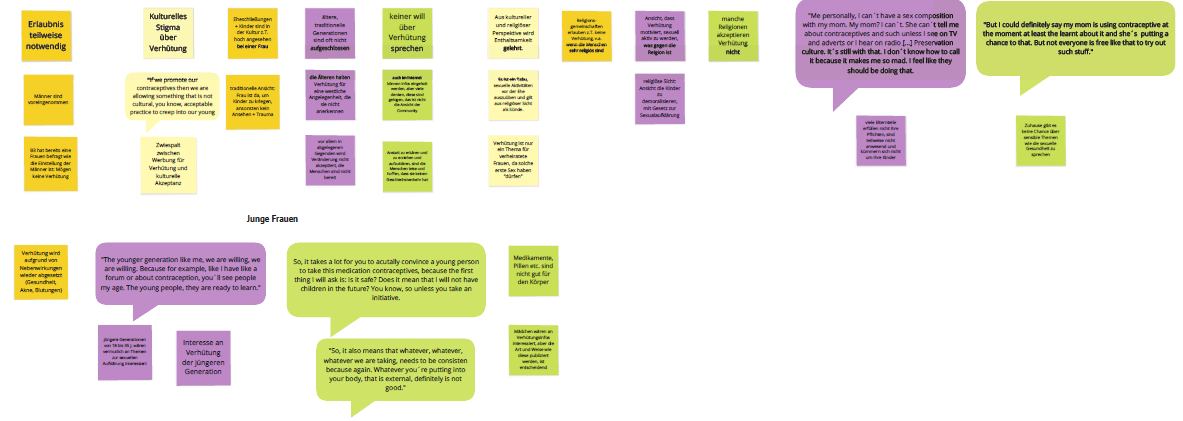


False Information Taboo


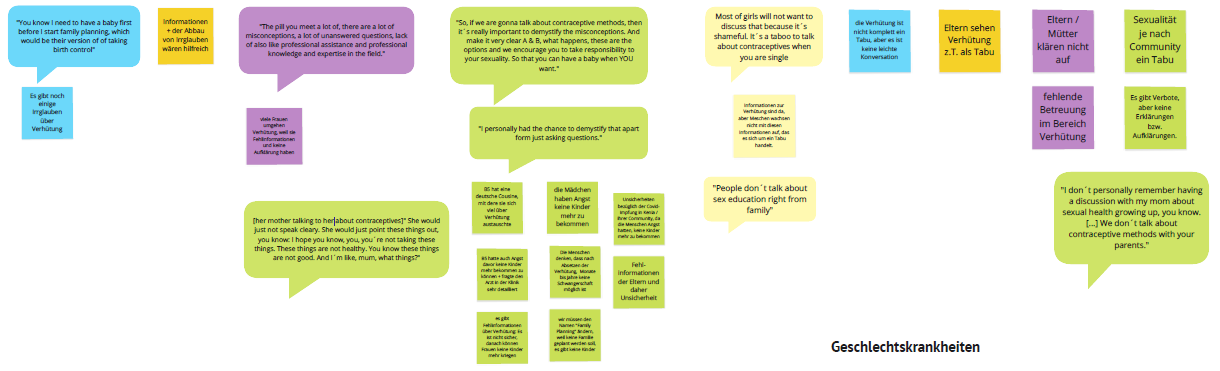


Sexual transmitted disease


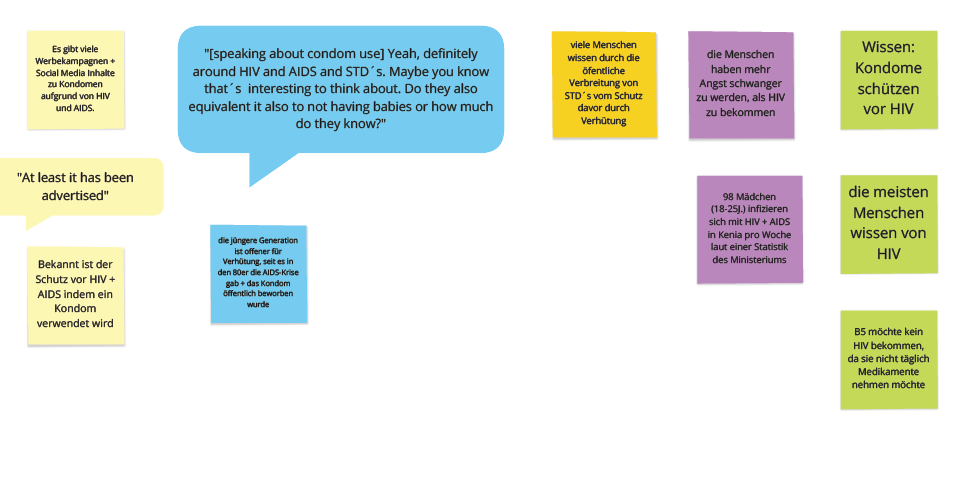


Source: Miroboard, own illustration

*Cluster 7: Technology Aspects*


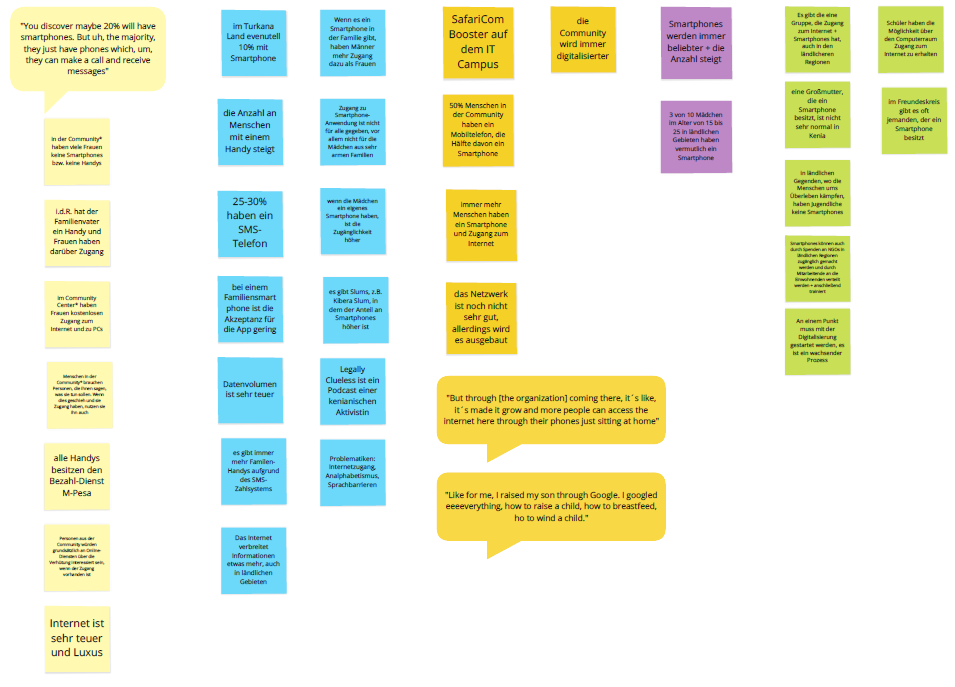


Source: Miroboard, own illustration

*Cluster 8: Application*

Recognition Implications Interaction


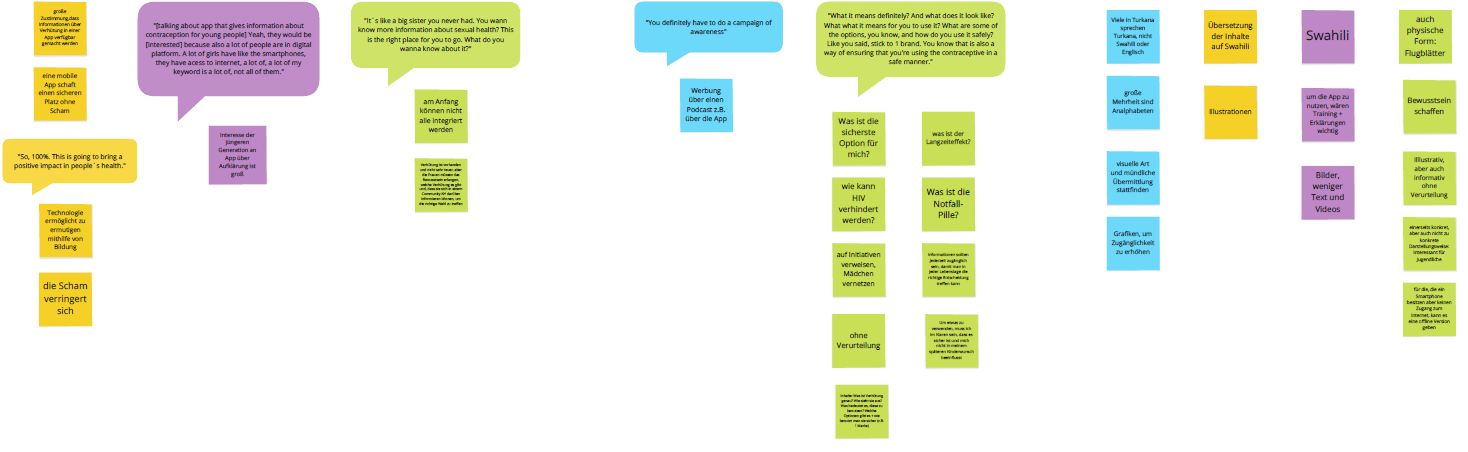


Source: Miroboard, own illustration

### *Figure S8 - Primary Persona – Ivy*


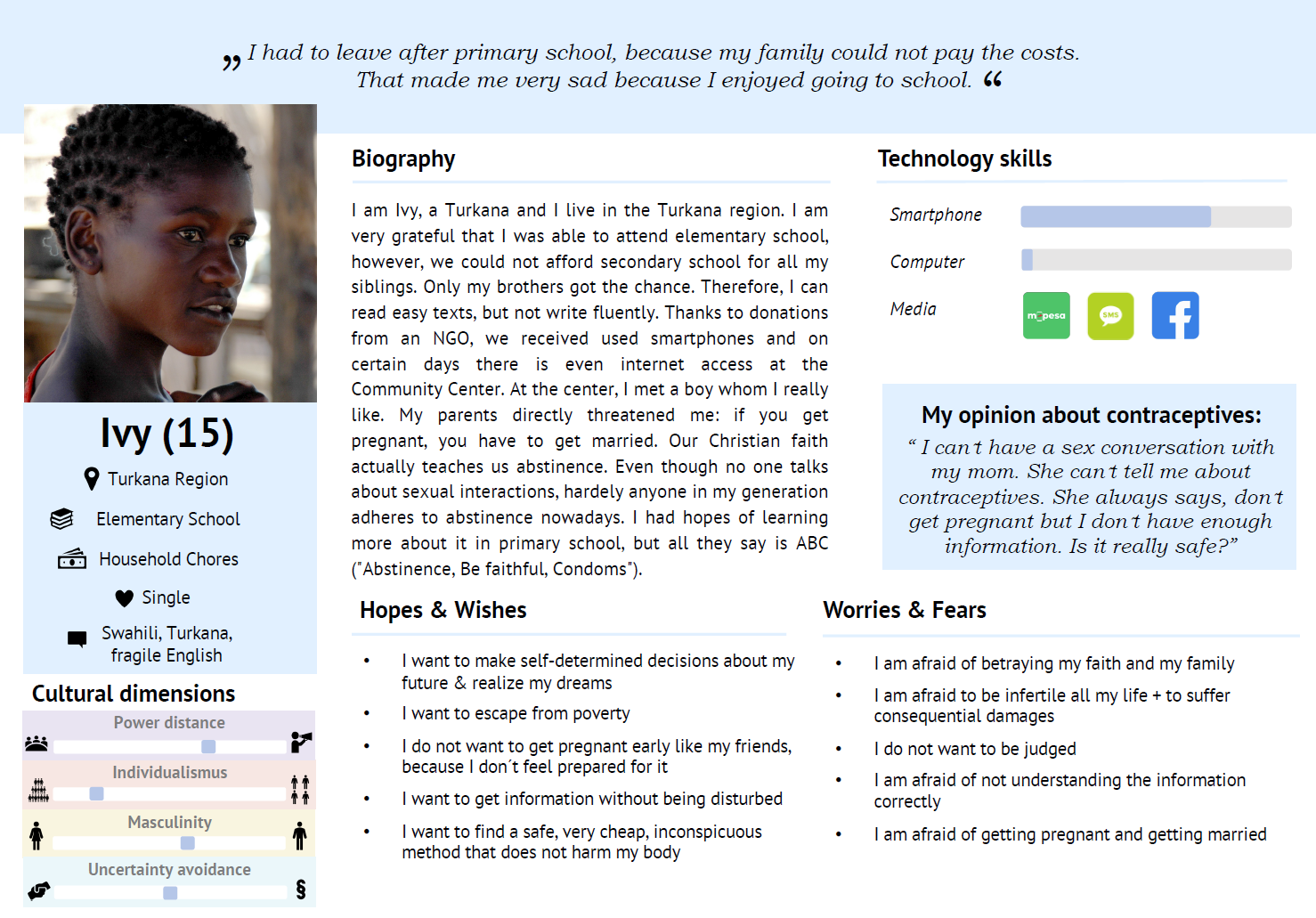


Source: Own illustration

*Figure S9 - Secondary Persona – Naomi*


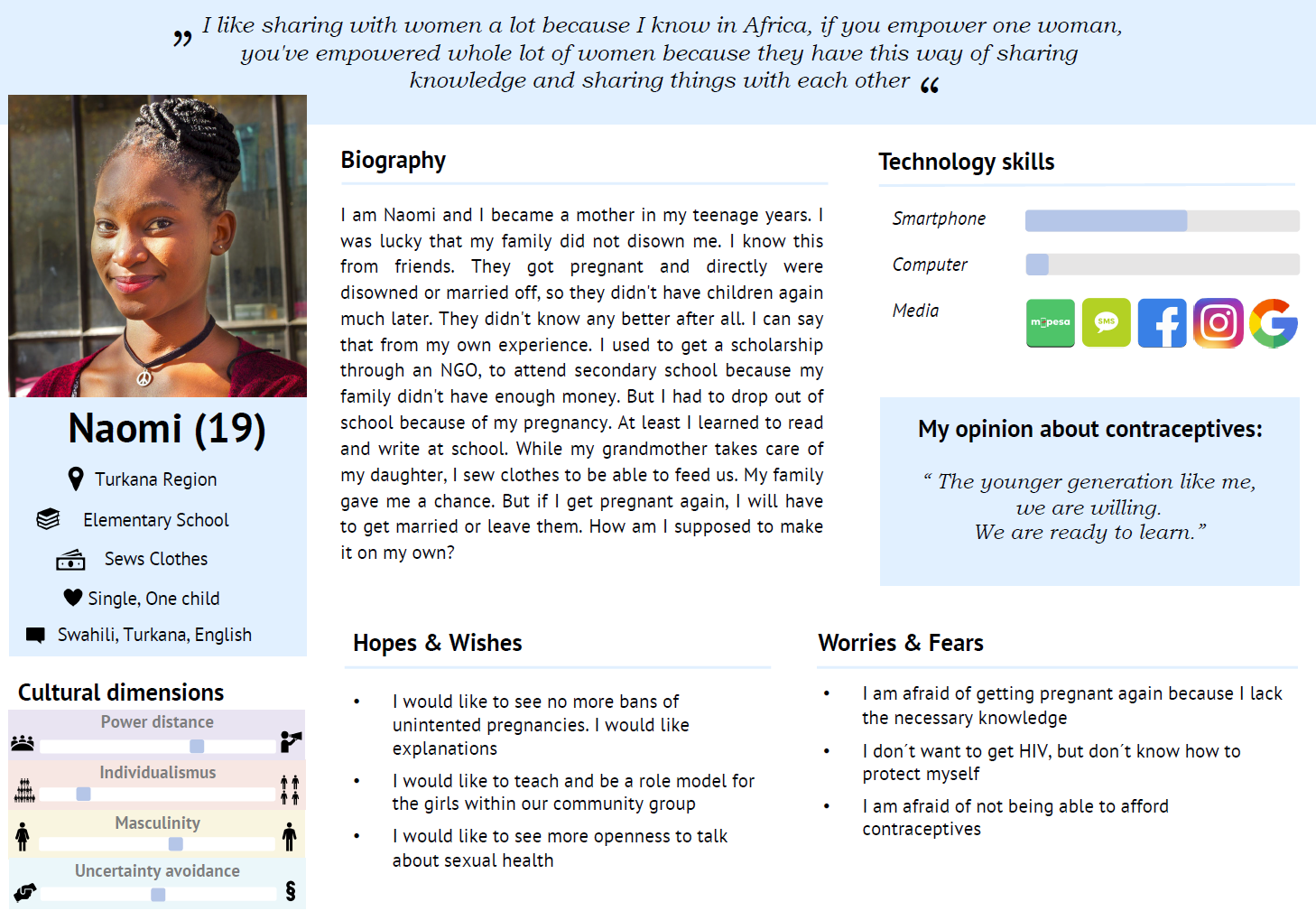


Source: Own illustration

Figure S10 - Anti-Persona – Zawadi


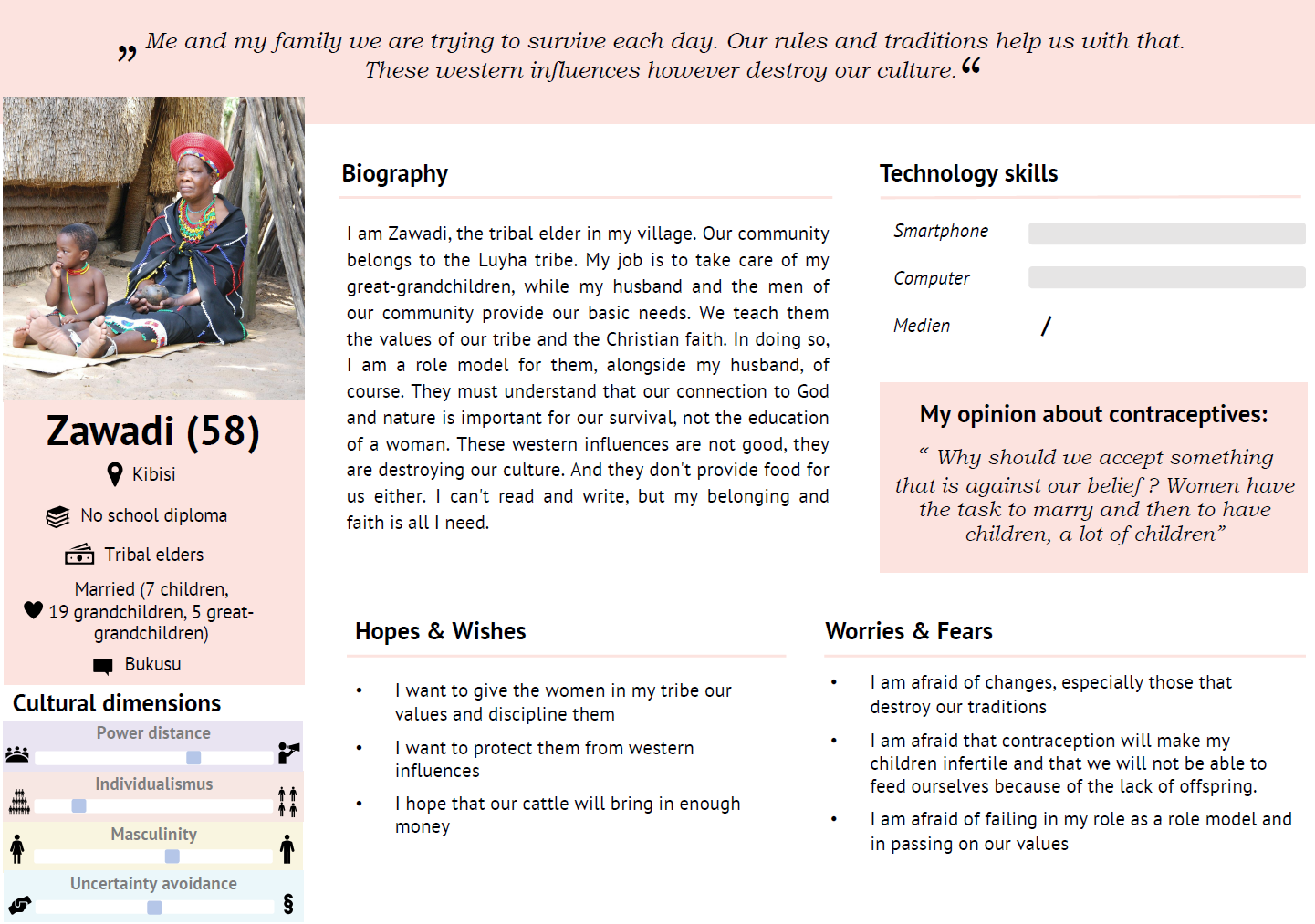
 Source: Own illustration

Figure S11 – Ivy Empathy Map


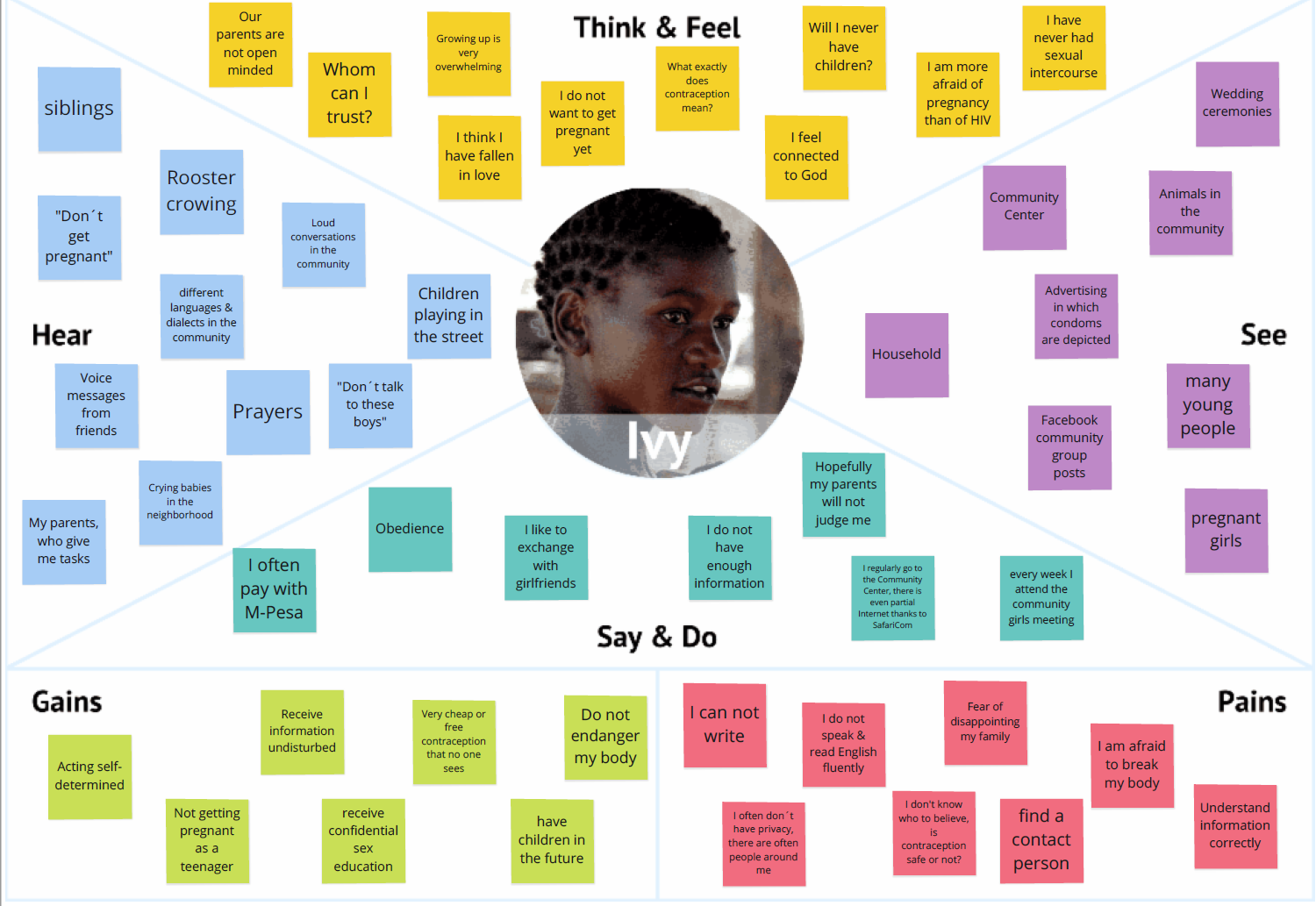


Source: Own illustration

Figure S12 - Naomi Empathy Map


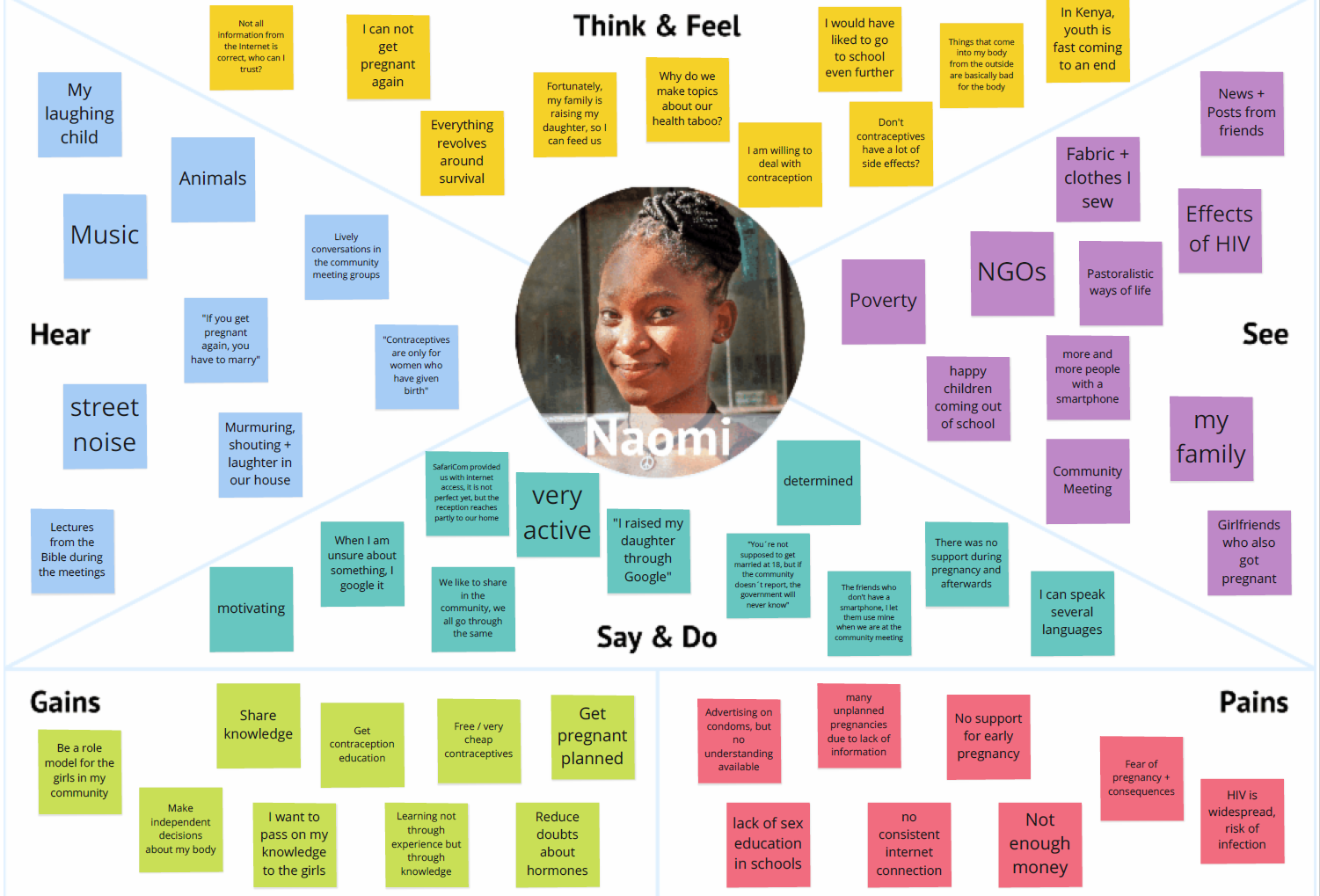


Source: Own illustration

Figure S13 – Zawadi Empathy Map


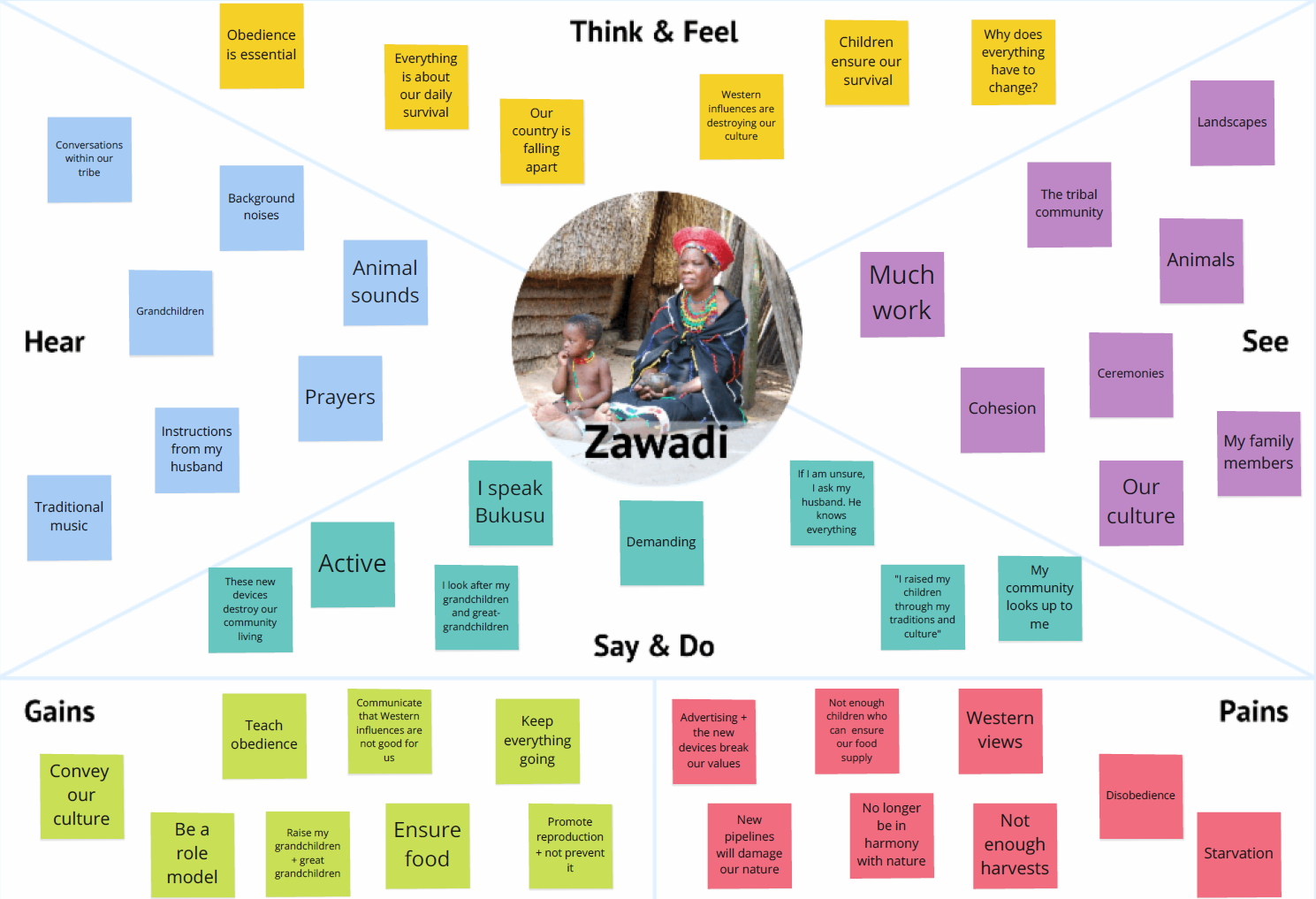


Source: Own illustration

Figure S14 – Ivy - Story Board


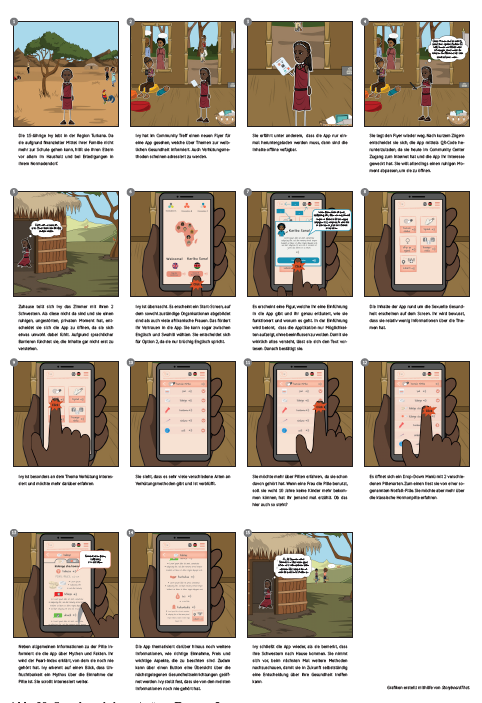


Source: Own illustration with StoryboardThat (Clever Prototypes, 2022)

Figure S15 – Naomi - Story Board


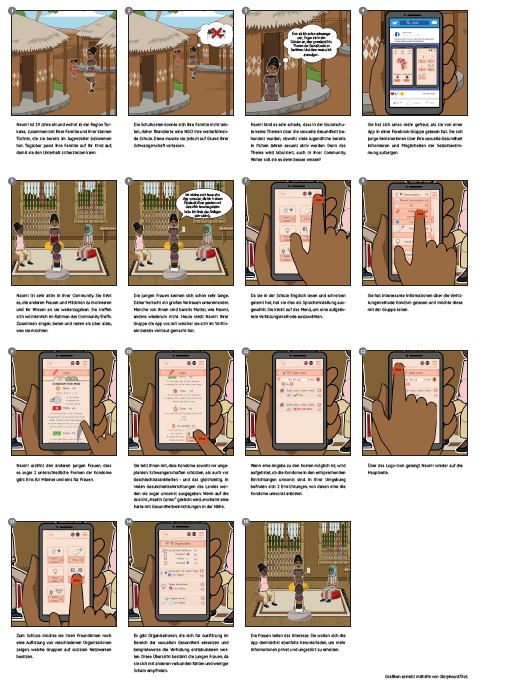


Source: Own illustration with StoryboardThat (Clever Prototypes, 2022
